# Interpeduncular GABAergic neuron function controls threat processing and innate defensive adaptive learning

**DOI:** 10.21203/rs.3.rs-4661779/v1

**Published:** 2024-09-20

**Authors:** Susanna Molas, Elora Williams, Leshia Snively, Benjamin O’Meara, Hannah Jacobs, Miranda Kolb, Rubing Zhao-Shea, Michael Baratta, Andrew Tapper

**Affiliations:** University of Colorado Boulder; University of Colorado Boulder; University of Colorado Boulder; University of Colorado Boulder; University of Colorado Boulder; University of Colorado Boulder; University of Colorado Boulder; University of Colorado Boulder; University of Massachusetts Medical School

## Abstract

The selection of appropriate defensive behaviors in the face of potential threat is fundamental to survival. However, after repeated exposures to threatening stimuli that did not signal real danger, an animal must learn to adjust and optimize defensive behaviors. Despite extensive research on innate threat processing, little is known how individuals change their defensive behaviors when presented with recurrent threat exposures without evidence of a real risk. Insight into this process is critical as its dysregulation may contribute to neuropsychiatric conditions, such as anxiety disorders. Here, we used the visual looming stimulus (VLS) paradigm in mice to investigate innate threat processing and adaptive defensive learning. Repeated exposure to VLS over consecutive sessions reduced immediate freezing responses and time spent inside a sheltered area upon VLS events, leading to an increase in foraging behaviors. Fiber photometry recordings and optogenetic manipulations revealed that VLS innate adaptive defensive learning is associated with reduced recruitment of the midbrain interpeduncular nucleus (IPN), a structure associated with fear and anxiety-related behaviors. Functional circuit-mapping identified a role for select IPN projections to the laterodorsal tegmental nucleus in gating defensive learning. Finally, we uncovered a subpopulation of IPN neurons that express the neuropeptide somatostatin and encode safety- and avoidance signals in response to VLS. These results identify critical behavioral signatures of innate defensive responses and a circuit that regulates the essential features of threat processing.

## Introduction

Individuals select optimal defensive strategies, such as escape or freezing, to avoid danger when threat is present (1, 2). Nevertheless, with repeated exposures to a potential threat without evidence of an aversive outcome, defensive behaviors must undergo adaptive learning, thus contributing to efficient action selection. Numerous neuropsychiatric conditions, including anxiety disorders, manifest maladaptation of threat responses (3), highlighting the importance of understanding these basic neurobehavioral processes.

Exposure to an overhead dark visual looming stimulus (VLS) naturally elicits innate defensive responses across multiple species, from fish to humans (1, 4). In rodents, exploring an open area supplied with a nest as a shelter, the detection of an expanding VLS to the upper visual eld triggers an immediate defensive behavior of freezing and escaping towards a sheltered area, seeking protection (5). Whether rodents shift their defensive responses to optimal behavioral sequences after repeated VLS and the mechanisms that support this innate defensive adaptive learning remain largely unknown.

Processing sensory information and coordinating appropriate motor output for defensive behaviors requires complex neural circuits (6, 7). Rodent and human studies have implicated an emerging circuit, arising in the habenula (Hb), that contributes to threat-related behaviors (8). The habenula nuclei play a crucial role in regulating emotional, motivational, and cognitive behaviors (9–12). Specifically, the medial part of the habenula (mHb) sends descending projections almost exclusively to the interpeduncular nucleus (IPN) of the midbrain (13), an axis implicated in anxiety (14–21) and fear responses (22–25). The IPN is highly enriched in GABAergic neurons that establish reciprocal connections with regions involved in motivation and affective behaviors, including the raphe, locus coeruleus, and laterodorsal tegmental nucleus (LDTg)(26, 27). Recent work indicates that the habenulo-interpeduncular axis, and IPN projections to the LDTg, mediate aversive and reward-related behaviors (28, 29). Yet, whether the IPN and associated neuronal circuits are recruited by VLS to adjust defensive behaviors has never been explored.

## Materials and methods

### Animals

All experiments followed the guidelines provided by the National Research Council with approved animal protocols from the Institutional Animal Care and Use Committee of the University of Massachusetts Chan Medical School. C57Bl/6J (Stock #000664, Jackson), *GAD2^Cre^* (Stock #10802, Jackson) and *Sst^Cre^* (Stock #013044, Jackson) mice were used. *Cre* lines were crossed with C57Bl/6J mice and only heterozygous animals used. All experiments included male mice. IPN Sst ablation experiments included males and females. Subject mice were kept under a reverse 12h light/dark cycle (lights ON at 7P.M.) for 3–4 weeks with ad *libitum* access to food and water, and individually housed for at least 5 days before any behavioral testing. Experiments were performed during the dark cycle phase (8A.M. to 5P.M.).

### Viral preparations

Biosensors, optogenetic and control plasmids packaged into viral particles were purchased from Addgene. For fiber photometry experiments we used pAAV.CAG.Flex.GCaMP6m.WPRE.SV40 (#100839-AAV5, 2.6×10^13^GC/ml), pGP.AAV.CAG.Flex.-jGCaMP7s.WPRE (#104495-AAVrg, 1.1×10^13^GC/ml). For tracing and optogenetic experiments we used pAAV.hSyn.DIO.EGFP (#50457-AAV5, 1.3×10^13^GC/ml and - AAVrg, 1.4×10^13^GC/ml), pAAV.hSyn.DIO.mCherry (#50459-AAV5, 1.8×10^13^GC/ml and - AAVrg, 1.5×10^13^GC/ml), pAAV.Ef1a.DIO.eNpHR3.0.EYFP (#26966-AAV5, 3.8×10^12^GC/ml), pAAV.Ef1a.double oxed.hChR2(H134R).mCherry.WPRE.HGHpA (#20297-AAV5 1.2×10^13^GC/ml) and pAAV. ex.taCasp3-TEVp (#45580-AAV5, 2.5×10^13^GC/ml). The viral stock pAAV.Ef1a.DIO.-eNpHR3.0.EYFP (#AV9115-rAAV2, 5.8×10^12^VM/ml) was obtained from UNC GTC Vector Core.

### Stereotaxic surgeries

Surgeries were performed under aseptic conditions as previously described (21). Mice (6–8 weeks old) were deeply anaesthetized using 100 mg/kg ketamine (VEDCO) and 10 mg/kg xylazine (LLOYD) and placed on a stereotaxic frame (Stoelting Co.). Viral solutions were microinjected at a controlled rate of 50 nl/min using a gas-tight 33G 10-μl neurosyringe (1701RN; Hamilton). Injection coordinates were (in mm, anteroposterior, mediolateral, dorsoventral and angle): IPN (− 3.4, − 0.5, − 4.86, 6°) and LDTg (−5.34, ± 0.4, −3.2, 0°). Viral volumes were 300 nl (IPN) and 300 nl/site (LDTg). For ber photometry and optogenetic experiments, 3–5 weeks post-viral injection, an optic ber implant (200-μm core diameter; 0.53N.A., Doric Lenses) held in a magnetic aluminum receptacle (Doric Lenses) was placed above the IPN and secured into the skull using adhesive (C&B Metabond cement, Parkell Inc.) followed by dental cement (Cerebond, PlasticsOne). Mice received IP injections of 1 mg/kg ketoprofen analgesic (Zoetis) and monitored for recovery. Animals showing no viral or off-target site viral expression or incorrect optic ber placement (< 10%) were excluded from analysis.

### Behavioral experiments

#### Visual looming stimulus (VLS) paradigm

The apparatus consisted of a rectangular Plexiglass maze (40×22×30 cm) with a projector screen (30×20 cm) above the arena and a rectangular shelter (10×12 cm) in one corner. All mice habituated to the apparatus and illumination settings for 8–10 min. After 24h, mice acclimated to the apparatus for 2–5 min before a VLS was randomly displayed from the screen while they actively explored the arena. Each VLS consisted of 15 consecutive dark expansions of 0.5s length and each mouse received 4–7 looming trials per day with a minimum inter-looming trial interval of 60s. The VLS test session was repeated for 3 consecutive days. For side-VLS, the screen was displayed from a wall view. The apparatus was cleaned between animals with 0.1% Micro-90 solution. A video-camera was used to record and track animals’ behavior using Ethovision XT (v15.0). The arena was subdivided in the nest area (12×10 cm), a safety zone adjacent to the nest (10×10 cm), a trigger zone where the VLS were displayed (12×12 cm) and a zone near the walls (5 cm). Early defensive responses included immediate freezing and maximum speed which were reported 2 sec upon VLS initiation, as well as latency to enter the nest and escape run (maximum speed 10 sec upon VLS). Late defensive responses included time spent inside the nest after the first nest entry once the VLS was presented, as well as time spent near the walls. The percent of time in each zone was estimated within 30 upon VLS initiation and averaged per each animal. Latency to and time in nest were manually scored by an experimenter blind to animals’ conditions.

#### Foot shock

Mice were habituated to a fear conditioning cage. GCaMP fluorescence from IPN neurons was recorded for 2 min before the rst shock. During a 15-min foot-shock period, ten shocks (0.5mA, 1-s duration) were delivered at random intervals and time-stamped into the photometry recording via a transistor-transistor logic (TTL) pulse from the fear conditioning system.

#### Open field

The apparatus consisted of an open-field chamber (42×38×30 cm). Each mouse was given 10 min to explore, and the time spent in the center and outer parts of the chamber was tracked from a video recording using Ethovision XT.

#### Elevated plus maze

The elevated plus maze (EPM) apparatus consisted of a central junction (5×5 cm) and had four arms elevated 45 cm above the floor with each arm positioned at 90° relative to the adjacent arms. Two closed arms were enclosed by high walls (30×5×15 cm) and the open arms were exposed (30×5×0.25 cm). All mice were given 5 min of free exploration.

#### Tail lift

Animals were picked up by their tails by an experimenter while they were actively exploring the home cage.

#### Fiber photometry and data analysis

Florescent signals from biosensors were recorded using a Doric Instruments Fiber Photometry System as previously described (28). A LED driver delivered excitation light at 465 nm and at 405 nm (~ 30–60μW output at the ber tip). The light was reflected into a 200μm 0.53N.A. optic fiber patch cord via the Dual Fluorescence Minicube. Emissions were detected with a femtowatt photoreceiver (Model 2151, Newport). Sampling (12 kHz) and lock-in demodulation of the fluorescence signals were controlled by Doric Neuroscience Studio software with a decimation factor of 50. A behavioral camera synchronized the photometry recordings with time-locked behavioral tracking systems.

Fiber photometry data analysis was performed using custom-written Matlab scripts. The 405 nm channel was scaled to the 465 nm by applying a least mean squares linear regression. Scaled signals were used to calculate the ΔF/F_0_ where ΔF/F_0_ = (465nm signal – fitted 405nm signal)/ fitted 405nm signal. Z-scores were calculated using the average baseline of ΔF/F_0_ values from the − 1.0 sec prior to the onset of VLS (considered as time zero, t = 0). The max and mean z-score were estimated between t = 0 and 10 sec upon VLS and averaged per animal. The min z-score for nest entry was estimated between t = 0 (nest entry) and 10 sec upon nest entry and averaged per animal. For IPN Sst^+^ GCaMP recordings, the max z-scores were determined − 2 to 2 sec time-locked to nest entry.

#### Optogenetics

Optic fiber implants were connected to a patch cable (Doric Lenses) and a commutator (rotary joint; LEDFRJ-B_FC for blue light and LEDFRJ-A_FC for yellow light, Doric Lenses), by means of an FC/SMC adapter to allow unrestricted movement, as previous reported (30). Mice habituated for 8–10 min to the VLS apparatus without receiving light photostimulation. On day 1 of VLS test, mice freely explored the apparatus for 2–5 min before VLS were displayed. A high-power LED driver (DC2200, Thorlabs) was used to generate light pulses time locked to VLS events at intensity ~ 2–5 mW at the fiber tip. Light photoinhibition (593nm, constant light) was delivered in time-locked mode by an experimenter blind to animals’ conditions, 2 sec prior, during and 2 sec post each VLS event. On day 2 of the test, mice were subjected to the VLS paradigm with no light delivery. Day 3 followed the same light stimulation protocol as day 1. For optogenetic photostimulation, light pulses (473nm, 20Hz, 12ms pulse, 3s) were delivered at intervals > 90s on days 1 and 3. No VLS was displayed. All sessions were video recorded from above (HDR-CX4440 camera, SONY) and computationally analyzed with Ethovision XT.

#### Immunostaining and microscopy

Immunohistochemistry and microscopy were performed as described previously (30). Mice received sodium pentobarbital (200 mg/kg) and transcardially perfused with ice-cold 0.1 M phosphate buffer saline (PBS, pH7.4) followed by 10 ml of cold 4% (W/V) paraformaldehyde (PFA). Brains were post-fixed in 4% PFA before transferred to 30% sucrose. Coronal sections (25 μm) were obtained using a freezing microtome (HM430; Thermo Fisher Scientific, MA, USA). Brain sections were permeabilized with 0.5% Triton X-100 (Sigma) for 10 min, blocked with 5% donkey serum (DS, Sigma) for 30 min and incubated with the primary antibody (rabbit anti-Sst, 1:700, sc-13099) overnight at 4 ºC. Slices were incubated in secondary antibody for 2 h (1:800; Life Technologies; donkey anti-rabbit 594, R37119). Nuclei were counterstained with DAPI. Viral expression was visualized using the endogenous fluorescence of the virus. All slices were imaged using a fluorescent microscope (Zeiss, Carl Zeiss MicroImmagine, Inc., NY, USA) connected to computer-associated image analyzer software (Axiovision Rel., 4.6.1).

#### Statistical analysiss

Data were analyzed by means of two-tailed unpaired *t*-test, one-way and two-way ANOVAs with/without repeated-measures (RM), or the restricted maximum likelihood (REML) mixed model, as indicated. Dunnett’s or Tukey’s *post hoc* tests were used for multiple comparisons. Two-tailed Pearson r was used for correlation analysis. Comparisons of z-scores photometry signals were made using the calculated average for each animal. Each data set was tested for normal distribution prior to analysis and presented as mean ± standard error of the mean (SEM). All statistical analyses were performed in GraphPad Prism 10.1.0. Software (Graphpad Software Inc.) and statistical significance was established at p < 0.05.

## Results

### Innate adaptive defensive learning: mice adjust behavioral response to a potential threat.

**Figure 1:**
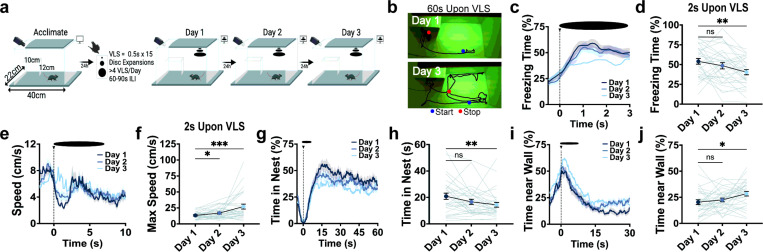
Mice exhibit innate defensive adaptive learning with repeated VLS exposures. **|** (**a**) Schematic of VLS paradigm. (**b**) Representative example of day 1 and day 3 animals’ tracked behavior 60s upon VLS. (**c**) Trace of freezing time (%) relative to VLS (t = 0), across 3 days. (**d**) Quantification of freezing time (%) 2 sec upon VLS in (c). One-way RM ANOVA (day effect: F_(2,92)_ = 7.082, *P* = 0.0025). Dunnett’s multiple comparisons **p < 0.01. (**e**) Trace of speed (cm/s) relative to VLS (t = 0), across 3 days. (**f**) Quantification of max speed (cm/s) 2 sec upon VLS in (e). One-way RM ANOVA (day effect: F_(2,92)_ = 10.74, *P* = 0.0014). Dunnett’s multiple comparisons *p < 0.05, ***p < 0.001. (**g**) Trace of time spent inside the nest (%) relative to VLS (t = 0), across 3 days. (**h**) Quantification of time in nest (s) after VLS across days. One-way RM ANOVA (day effect: F_(2,92)_ = 6.248, *P* = 0.0037). Dunnett’s multiple comparisons **p < 0.01. (**i**) Trace of time spent near the wall (%) relative to VLS (t = 0), across 3 days. (**j**) Quantification of time spent near the wall (%), 30 sec upon VLS in (i). One-way RM ANOVA (day effect: F_(2,92)_ = 4.713, *P* = 0.0165). Dunnett’s multiple comparisons *p < 0.05. (n = 31 mice). Data represent mean ± SEM.

We implemented the VLS paradigm to investigate an animal’s capacity to adjust innate defensive behaviors in the absence of a real risk (Fig. 1a). Mice were placed in a plexiglass apparatus that contained a rectangular nest in one corner and a projector screen that displayed a VLS while animals actively explored the arena. The detection of a VLS from above, but not from a side view (Supplementary Fig. 1a-c), triggered an immediate defensive response of freezing followed by running to the nest (here de ned as early responses to VLS). Animals also spent a significant amount of time in the confines of the shelter, presumably avoiding potential threat, before engaging in exploratory behaviors (here de ned as late responses to VLS). However, with multiple exposures of an overhead VLS for 3 consecutive days, without evidence of aversive outcomes, mice learned to adjust both early and late VLS-evoked innate defensive strategies (Fig. 1b-j). Across the 3 days, immediate freezing significantly reduced (Fig. 1c-d), whereas speed increased (Fig. 1e-f). Mice continued to run with similar maximum speed (10 sec upon VLS) and exhibited similar escape latencies to the nest in response to repeated VLS over the 3 days (Supplementary Fig. 1d-e). To measure late VLS-induced defensive behaviors, we tracked the animal’s position throughout arena zones (Supplementary Fig. 1f and methods). The amount of time spent inside the nest upon VLS exposure decreased over repeated days (Fig. 1g-h), while animals shifted to more exploratory behavior near the walls (Fig. 1i-j). We did not observe adaptive changes with time spent in the trigger zone, where the VLS is presented (Supplementary Fig. 1g-h), or time in the safety zone adjacent to the nest (Supplementary Fig. 1i-j). Notably, adjustment of defensive responses was not detected within a single-day trial session (Supplementary Fig. 2), suggesting that optimization of defensive strategies may reflect learning and consolidation processes.

We performed linear correlation analysis to further investigate threat-evoked innate adaptive defensive learning with repeated VLS (Supplementary Fig. 3 and Supplementary Tables 1–6). For early responses to VLS, we found that latency to the nest showed a strong negative correlation with the maximum speed the animals reached 10 sec upon VLS, which was maintained across the 3 sessions, indicating the faster the animals ran, the earlier they entered the nest. Interestingly, the early response of freezing upon VLS initiation predicted late defensive responses such as the total amount of time the animals would spend inside the nest. Time spent in the nest was also positively correlated with escape including latency to the nest and maximum speed 10 sec upon VLS only on day 1, but not in later sessions. These results suggest that escape behaviors (i.e. running to the nest) and avoidance behaviors (i.e. time spent inside the nest) are related to each other at initial sessions but may become more dissociable once the animals learn to adjust defensive responses.

### Exposure to potential threat engages activity of IPN GAD2 neurons that adjusts with defensive learning.

**Figure 2:**
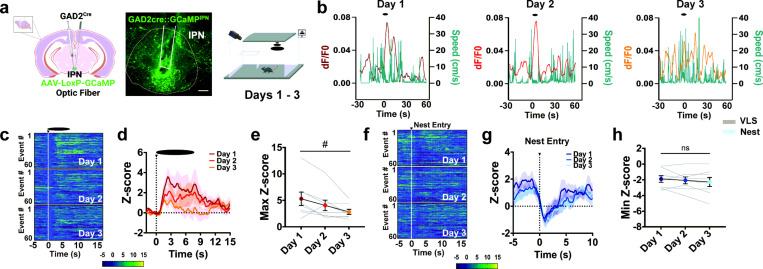
IPN GABAergic neurons respond to VLS and adapt with multiple exposures. **|** (**a**) *Left*, schematic and representative image of GCaMP injection and fiber placement in the IPN in *GAD2 Cre* mice. Scale bar 100μm. *Right*, schematic of VLS paradigm. (**b**) Example of speed trace (cm/s) compared to time-locked IPN *GAD2* fiber photometry signals (dF/F0) relative to VLS (t = 0), across 3 days. Heatmap representations (**c**) and z-score values (**d**) of time-locked IPN *GAD2* neuronal activity relative to VLS (t = 0), across 3 days. (**e**) Quantification of responses in (d) as maximum z-score values detected 10 sec upon VLS. One-way RM ANOVA (day effect: F_(2,23)_ = 5.061, *P* = 0.0328). # p < 0.05. Heatmap representations (**f**) and z-score values (**g**) of time-locked IPN *GAD2* neuronal activity relative to the time of nest entry (t = 0), across 3 days. (**h**) Quantification of activity in (f) as minimum z-score values detected 10 sec upon nest entry. One-way RM ANOVA (day effect: F_(2,23)_ = 1.348, *P* = 0.2916). (n = 8 mice). Data represent mean ± SEM.

To study the neurocircuitry behind threat adaptation and defensive learning we focused on the IPN of the midbrain, an emerging region associated with anxiety and fear (31). The IPN is an inhibitory nucleus highly enriched in GABAergic neurons that respond to aversive stimuli (32–34). We combined *in vivo* fiber photometry recordings with mouse behavior to test if IPN GABAergic neuronal activity is engaged by VLS. Specifically, we expressed *Cre*-dependent GCaMP in the IPN of mice driving *Cre* recombinase under the control of the glutamic acid decarboxylase 2 enzyme promoter (*GAD2^Cre^* mice) and recorded IPN activity dynamics time-locked to VLS events (Fig. 2a). In mice presented with an overhead VLS, we detected a significant increase in IPN *GAD2* neuronal activity that was absent in control mice expressing *Cre*-dependent eGFP in the IPN instead of GCaMP or if the same VLS was presented from a side view (Supplementary Fig. 4a-b). Additional aversive stimuli, including a tail lift or a foot shock, also raised the activity of the IPN *GAD2* neuronal population (Supplementary Fig. 4c-d). Remarkably, IPN neuronal responses to the VLS decreased from day 1 to day 3, as mice learned to optimize innate defensive strategies (Fig. 2b-e). Other behaviors, such as rearing against the wall, also increased IPN activity. However, the IPN GABAergic activity during rearing remained stable across the 3 days (Supplementary Fig. 4e), providing evidence that the decrease in signal was not due to photobleaching. Reductions of VLS-evoked IPN *GAD2* neuronal activation across days were not detected within a trial session (Supplementary Fig. 4f-g).

Interestingly, IPN *GAD2* neuronal activity dynamics inversely mirrored changes in speed during VLS events (Fig. 2b), as well as across the whole VLS session (Supplementary Fig. 5a). Correlation analysis demonstrated VLS-induced activation levels of IPN *GAD2* neurons negatively related with speed 10 sec post-VLS initiation (Supplementary Fig. 5b). Furthermore, VLS-induced engagement of IPN *GAD2* neurons positively correlated with latency to nest (Supplementary Fig. 5c) and time spent near the walls (Supplementary Fig. 5d). Notably, we also detected reduced activity of IPN GABAergic neurons when mice entered the nest (Fig. 2f-h), although these signals did not adjust across repeated sessions. Altogether these results suggested the dynamics of IPN *GAD2* neuronal activity re ected different aspects of VLS evoked defensive actions.

### Silencing activity of IPN GAD2 neurons during VLS presentations reduces innate defensive behaviors.

**Figure 3:**
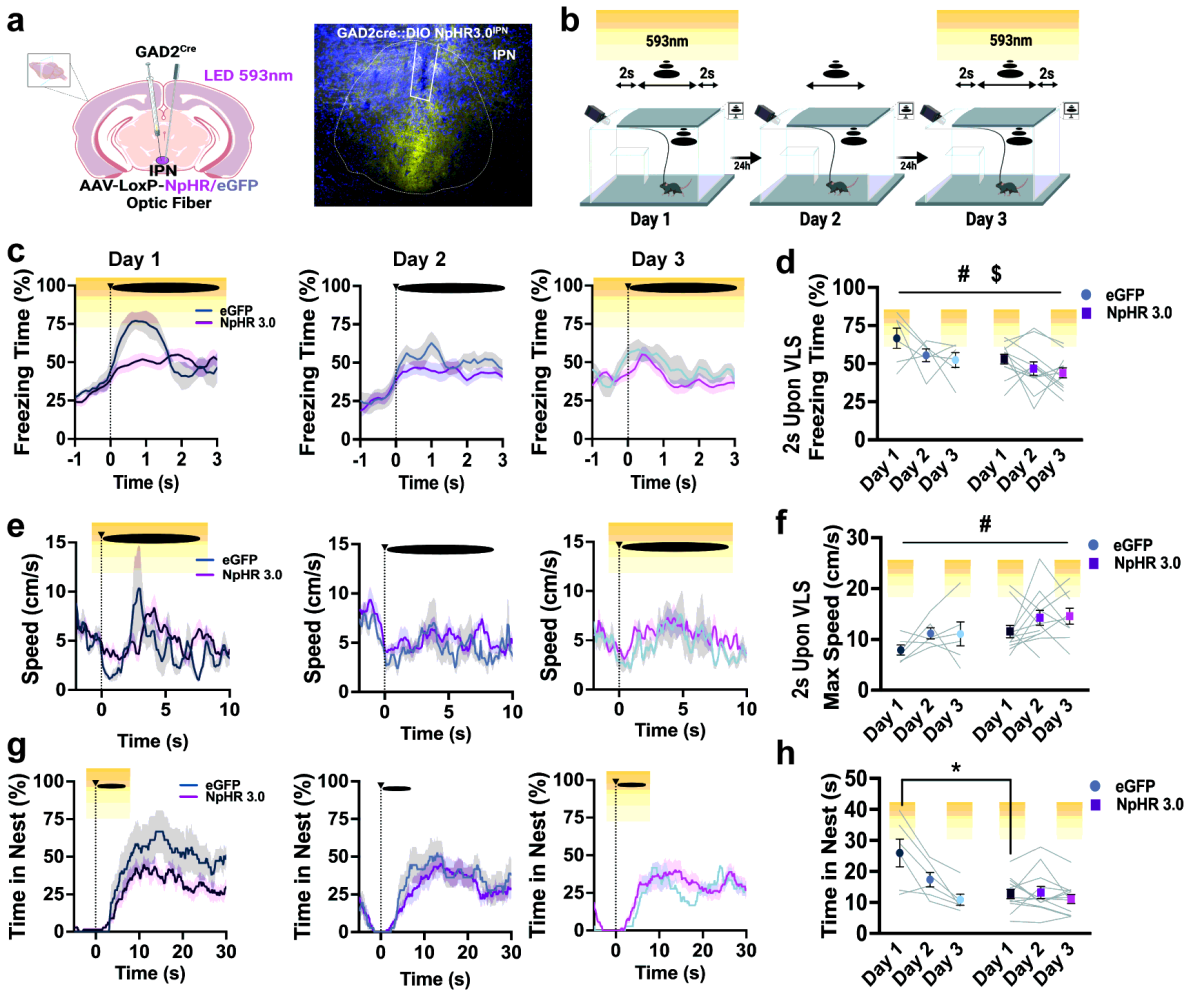
Photoinhibition of IPN GABAergic neurons reduces early and late defensive behaviors. **|** (**a**) Schematic and representative image of NpHR3.0 injection and fiber placement in the IPN in *GAD2 Cre* mice. Scale bar 100μm. (**b**) Schematic representation of 3-day VLS paradigm with NpHR inhibition on days 1 and 3 (yellow). (**c**) Traces of freezing time (%) relative to VLS (t = 0), across 3 days in IPN *GAD2* eGFP and NpHR animals. (**d**) Quantification of freezing time (%) 2 sec upon VLS in (c). Two-way RM ANOVA (day effect: F_(2,32)_ = 3.928, *P* = 0.0312; treatment effect: F(_1,16_) = 6.679, *P* = 0.0200; interaction: F_(2,32)_ = 0.2304, *P* = 0.7956), treatment effect # p < 0.05, day effect $ p < 0.05. (**e**) Traces of speed (cm/s) relative to VLS (t = 0), across 3 days in IPN *GAD2* eGFP and NpHR animals. (**f**) Quantification of max speed (cm/s) 2 sec upon VLS in (e). Two-way RM ANOVA (day effect: F_(2,32)_ = 2.299, *P* = 0.1185; treatment effect: F_(1,16)_ = 6.514, *P* = 0.0213; interaction: F_(2,32)_ = 0.0294, *P* = 0.9711), treatment effect # p < 0.05. (**g**) Trace of time spent inside the nest (%) relative to VLS (t = 0), across 3 days in IPN *GAD2* eGFP and NpHR animals. (**h**) Quantification of time in nest (s) after VLS across days. Two-way RM ANOVA (day effect: F_(2,32)_ = 17.02, P < 0.0001; treatment effect: F_(1,16)_ = 4.336, *P* = 0.0537; interaction: F_(2, 32)_ = 11.45, *P* = 0.0002), Tukey’s multiple comparisons *p<0.05. (n = 6 eGFP and 12 NpHR3.0 mice). Data represent mean ± SEM.

To determine the functional implication of IPN *GAD2* neurons in defensive responses, we used optogenetics and selectively silenced these neurons during VLS events. *GAD2^Cre^* mice were injected with *Cre*-dependent halorhodopsin (NpHR) or eGFP control virus and implanted with an optic fiber in the IPN (Fig. 3a). Animals then underwent the 3-day VLS paradigm with photoinhibition 2 sec prior to VLS, which remained ON until 2 sec post-VLS, on days 1 and 3 (Fig. 3b). Photoinhibition of IPN GABAergic neurons reduced both early and late defensive responses. Compared to the controls, IPN NpHR animals exhibited a decrease in VLS-induced immediate freezing response (Fig. 3c-d) along with increased speed upon VLS display (Fig. 3e-f). Additionally, silencing IPN *GAD2* neurons led to decreased time spent in the nest on day 1 of the looming session (Fig. 3g-h); instead, animals remained in the vicinity of the safety zone (Supplementary Fig. 6a-b). Other defensive responses including latency to enter the nest (Supplementary Fig. 6c), time spent near the walls (Supplementary Fig. 6d-e), or in the trigger area (Supplementary Fig. 6f-g) were not significantly affected by IPN *GAD2* photoinhibition. Overall, these data suggest that IPN GABAergic neurons mediate both early and late VLS-evoked defensive behaviors, particularly on the first exposure session when neurons are highly engaged by VLS.

To complement these experiments, we also asked if activating IPN GABAergic neurons is enough to elicit changes in innate defensive strategies. To this aim, we injected the IPN of *GAD2^Cre^* mice with *Cre*-dependent channelrhodopsin (ChR2) and implanted an optic ber in the target site (Supplementary Fig. 7a). In this experiment, no VLS was presented; instead, animals received IPN *GAD2* neuronal photostimulation when they entered the trigger zone, on days 1 and 3 (Supplementary Fig. 7b). Optogenetic excitation alone did not produce differences between groups for the defensive responses measured (Supplementary Fig. 7c-k).

### IPN GAD2 neurons projecting to the LDTg are engaged by VLS and reduce activation with innate defensive adaptive learning.

**Figure 4:**
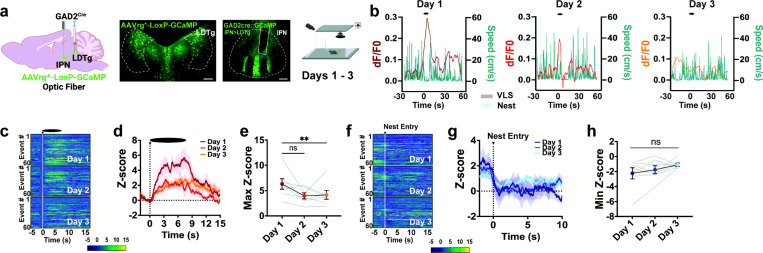
IPN GAD2 neurons projecting to the LDTg are engaged by VLS and adapt with multiple exposures. **|** (**a**) *Left*, schematic of retrograde viral injection and optic fiber strategy used. *Middle*, representative images of retroviral mediated GCaMP injection in the LDTg and fiber placement in the IPN in *GAD2 Cre* mice. Scale bar 100μm. *Right*, schematic of VLS paradigm. (**b**) Example of speed trace (cm/s) compared to time-locked IPNàLDTg *GAD2* fiber photometry signals (dF/F0) relative to VLS (t = 0), across 3 days. Heatmap representations (**c**) and z-score values (**d**) of time-locked IPNàLDTg *GAD2* neuronal activity relative to VLS (t = 0), across 3 days. (**e**) Quantification of responses in (d) as maximum z-score values detected 10 sec upon VLS. One-way RM ANOVA (day effect: F_(2,20)_ = 5.409, *P* = 0.050), Dunnett’s multiple comparisons. ** p < 0.01. Heatmap representations (**f**) and z-score values (**g**) of time-locked IPNàLDTg *GAD2* neuronal activity relative to the time of nest entry (t = 0), across 3 days. (**h**) Quantification of activity in (f) as minimum z-score values detected 10 sec upon nest entry. One-way RM ANOVA (day effect: F_(2,20)_ = 1.856, *P* = 0.2059). (n = 7 mice). Data represent mean ± SEM.

The IPN *GAD2* neuronal population consists of projection neurons that innervate brain regions involved in affective and motivational behaviors, including the raphe and the LDTg (26, 27). Here, we hypothesize the IPN may use the LDTg, an area previously associated with fear and anxiety (35), to convey innate defensive behaviors. To test if this circuit responded to the VLS, we recorded activity dynamics in *GAD2^Cre^* mice bilaterally injected with a retrogradely-transported AAVrg *Cre*-dependent GCaMP into the LDTg and implanted with an optic fiber in the IPN (Fig. 4a). The presentation of overhead VLS triggered an elevated activation of *GAD2*^IPN◊LDTg^ neurons that significantly decreased over consecutive sessions (Fig. 4b-e). No reductions in VLS-induced *GAD2*^IPN◊LDTg^ circuit engagement were detected within a trial session (Supplementary Fig. 8a-b). Similarly to overall IPN *GAD2* neurons, activity dynamics of the *GAD2*^IPN◊LDTg^ circuit inversely mirrored changes in speed (Fig. 4b) and negatively correlated with speed levels 10 sec upon VLS initiation (Supplementary Fig. 8c). Additional analysis did not nd significant correlations between VLS-induced *GAD2*^IPN◊LDTg^ circuit engagement and latency to nest (Supplementary Fig. 8d), although *GAD2*^IPN◊LDTg^ activation positively predicted the time mice spent near the walls (Supplementary Fig. 8e). Akin to IPN *GAD2* measures, *GAD2*^IPN◊LDTg^ neurons showed a decrease in circuit activity when animals entered inside the nest that was sustained across the three sessions (Fig. 4f-h). This data demonstrates that VLS-related information engages the projection from the IPN to the LDTg area, which may participate in threat processing and adaptive defensive learning.

### Silencing IPN GAD2 neurons projecting to the LDTg occludes innate defensive adaptive learning.

**Figure 5:**
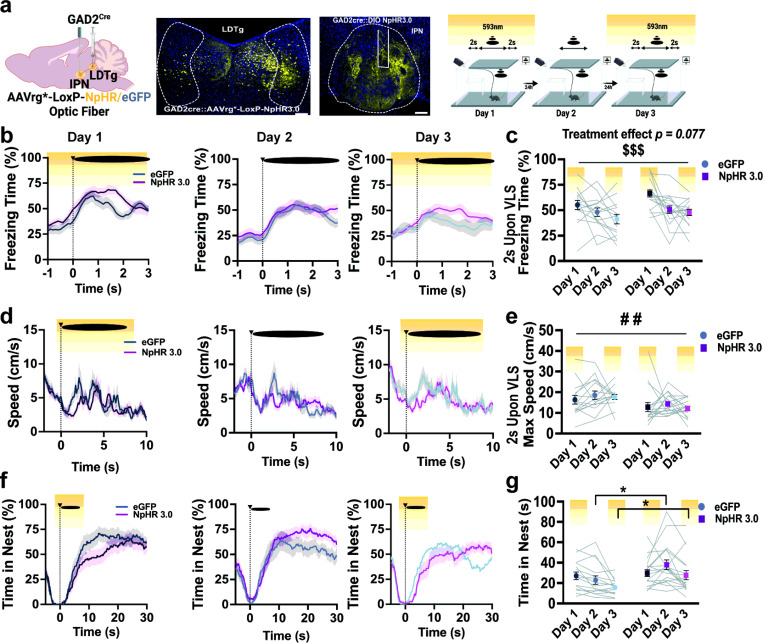
IPN→LDTg GAD2 neuronal circuit controls defensive adaptive learning **|** (**a**) *Left*, schematic and representative image of retrograde NpHR3.0 injection in the LDTg and fiber placement in the IPN in *GAD2 Cre* mice. Scale bar 100μm. *Right*, schematic representation of 3-day VLS paradigm with NpHR inhibition on days 1 and 3 (yellow). (**b**) Traces of freezing time (%) relative to VLS (t = 0), across 3 days in IPNàLDTg *GAD2* eGFP and NpHR animals. (**c**) Quantification of freezing time (%) 2 sec upon VLS in (b). Two-way RM ANOVA (day effect: F(_2,60_) = 11.16, *P* = 0.0001; treatment effect: F(_1,30_) = 3.338, *P* = 0.0777; interaction: F_(2,60)_ = 0.7984, *P* = 0.4548), day effect $ $ $ p < 0.001. (**d**) Traces of speed (cm/s) relative to VLS (t = 0), across 3 days in IPNàLDTg *GAD2* eGFP and NpHR animals. (**e**) Quantification of max speed (cm/s) 2 sec upon VLS in (d). Two-way RM ANOVA (day effect: F_(2,60)_ = 0.6815, *P* = 0.4984; treatment effect: F(_1,30)_ = 9.599, *P* = 0.0042; interaction: F_(2,60)_ = 0.1906, *P* = 0.8270). (**f**) Trace of time spent inside the nest (%) relative to VLS (t = 0), across 3 days in IPNàLDTg *GAD2* eGFP and NpHR animals. (**g**) Quantification of time in nest (s) after VLS across days. Two-way RM ANOVA (day effect: F_(2,60)_ = 6.927, *P* = 0.0020; treatment effect: F_(1,30)_ = 3.496, *P* = 0.0713; interaction: F_(2,60)_ = 3.192, *P* = 0.0481), Tukey’s multiple comparisons *p<0.05. (n = 15 eGFP and 17 NpHR3.0 mice). Data represent mean ± SEM.

To test if IPN GABAergic IPN neurons projecting to the LDTg are necessary for innate adaptive defensive responses, we used closed-loop optogenetic approaches in a circuit-specific manner. To this aim, we injected a retrograde *Cre*-dependent AAVrg NpHR or eGFP into the LDTg and placed an opticfiber in the IPN (Fig. 5a). Animals underwent the same VLS paradigm with circuit photoinhibition time-locked to VLS events. Experimental and control mice demonstrated a significant reduction in the early defensive behavior of freezing across days (Fig. 5b-c). However, compared to the controls, optogenetic silencing of the IPN◊LDTg circuit slightly increased freezing behavior (Fig. 5b-c) and significantly reduced max speed 2 sec upon VLS events (Fig. 5d-e). Noticeably, control mice significantly reduced the time spent in the nest after VLS presentation across sessions, whereas mice with *GAD2*^IPN◊LDTg^ circuit photoinhibition continued spending a significant amount of time inside the nest over VLS days (Fig. 5f-g), suggesting that inhibition of this circuit occludes adaptive learning of innate threat responses. Time spent in other zones, such as the wall, safe, and trigger as well as latency to nest, were not influenced by optogenetic inhibition of the *GAD2*^IPN◊LDTg^ circuit (Supplementary Fig. 9).

### A subpopulation of IPN neurons expressing Sst encodes defensive responses in a threatening environment.

**Figure 6:**
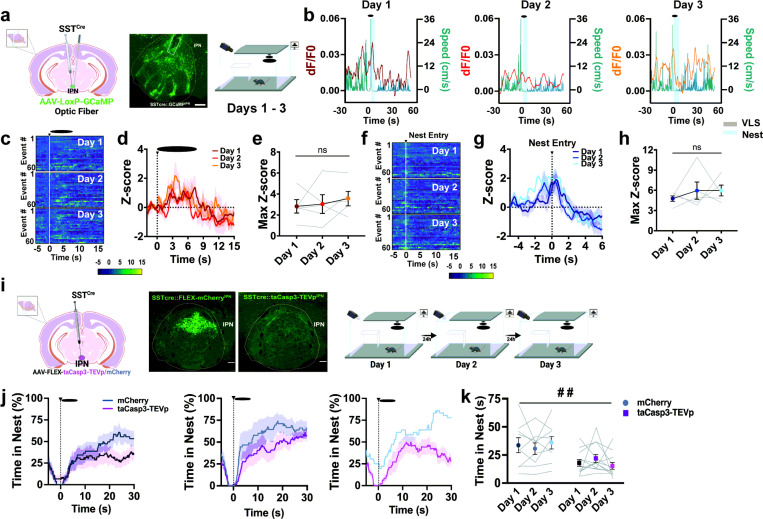
IPN Sst+ neurons encode threat and safety signals and control late defensive actions. **|** (**a**) *Left*, schematic of viral injection and fiber placement; representative image of viral-mediated GCaMP expression and fiber placement in IPN of Sst^Cre^ mice. Scale bar 100μm. *Right*, VLS paradigm. (**b**) Representative traces of IPN Sst^+^ activity recordings (dF/F0) compared to speed (cm/s) relative to VLS presentations (t=0), across days 1–3. Heatmap representations (**c**) and z-score values (**d**) of time-locked IPN Sst^+^ neuronal activity relative to VLS (t = 0), across 3 days. (**e**) Quantification of responses in (d) as maximum z-score values detected 10 sec upon VLS. One-way RM ANOVA (day effect: F_(2,14)_ = 0.4439, *P* = 0.6262). Heatmap representations (**f**) and z-score values (**g**) of time-locked IPN Sst^+^ neuronal activity relative to the time of nest entry (t = 0), across 3 days. (**h**) Quantification of activity in (g) as maximum z-score values detected during nest entry. One-way RM ANOVA (day effect: F_(2,14)_ = 0.5364, *P* = 0.5166). (n = 5 mice). (**i**) *Left*, Schematic of taCasp3-TEVp injection in the IPN in *Sst Cre* mice and representative image of *Sst* immunostaining (green) in the IPN of control and ablation animals. Scale bar 50μm. *Right*, Schematic representation of 3-day VLS paradigm. (**j**) Trace of time spent inside the nest (%) relative to VLS (t = 0), across 3 days in IPN Sst mCherry and taCasp3-TEVp animals. (**k**) Quantification of time in nest (s) after VLS across days. Two-way RM ANOVA (day effect: F_(2,38)_ = 0.0254, *P* = 0.9713; treatment effect: F_(1,19)_ = 8.818, *P* = 0.0079; interaction: F_(2, 38)_ = 1.858, *P* = 0.1698), treatment effect ## p < 0.01. (n = 9 mCherry and 12 taCasp3-TEVp mice). Data represent mean ± SEM.

Within the IPN, a GABAergic subpopulation expressing somatostatin (Sst)(36) demonstrates a highly selective dorso-rostral to ventro-caudal gradient (Supplementary Fig. 10a). Sst is a neuropeptide typically co-released with GABA and involved in anxiety-like behaviors (37). To explore whether IPN Sst^+^ responded to VLS, we injected *Cre*-dependent GCaMP in the IPN of mice expressing *Cre* recombinase under the control of the Sst promoter (Fig. 6a and Supplementary Fig. 10b). We found that aversive stimuli such as VLS presentations (Fig. 6b-e), a tail lift (Supplementary Fig. 10c) or a foot shock (Supplementary Fig. 10d), triggered an increase in IPN Sst^+^ neuronal activity. Remarkably, VLS-induced IPN Sst^+^ activation occurred after reaching max speed upon VLS (Fig. 6b) and did not reduce from day 1 to day 3 (Fig. 6c-e). Interestingly, we also detected IPN Sst^+^ activation time-locked to nest entry, although these signals did not adjust across repeated VLS sessions (Fig. 6f-h). Further analysis demonstrated on day 1 of the looming test, the engagement of IPN Sst^+^ neurons with nest entry positively correlated with speed levels 10 sec upon VLS (Supplementary Fig. 10e) and time spent inside the nest (Supplementary Fig. 10f), while it negatively correlated with nest latency (Supplementary Fig. 10g) or time spent near the walls (Supplementary Fig. 10h). Overall, IPN Sst^+^ activation with nest entry consistently predicted VLS-induced defensive behaviors, suggesting these neurons may encode safety and avoidance aspects of threat processing.

### Genetic ablation of IPN Sst + neurons reduces anxiety-like behaviors.

We next addressed the role of IPN Sst^+^ neurons in threat processing and defensive learning by genetically ablating this neuronal population using a *Cre*-dependent caspase 3 approach (Fig. 6i). Removing IPN Sst^+^ neuronal function did not affect VLS-induced freezing (Supplementary Fig. 11a-b) or changes in speed (Supplementary Fig. 11c-d) across the 3-day looming sessions. Nevertheless, we observed that IPN Sst^+^ ablated animals significantly reduced the time spent inside the nest as compared to mCherry control mice (Fig. 6j-k). In contrast, animals with IPN Sst ablation spent more time nearby the safety (Supplementary Fig. 11e-f) and wall zones (Supplementary Fig. 11g-h), without affecting nest latency (Supplementary Fig. 11i). The reduction in nest time observed by animals with ablated IPN Sst + neurons support the notation that these neurons play a role in processing avoidance-related aspects of threat-evoked defensive behavior, but not motor function. Along these lines, we also detected that IPN Sst^+^ ablation increased the time spent in the open arms of the elevated plus maze (Supplementary Fig. 11j) but not the number of closed arm entries (Supplementary Fig. 11k). Similarly, IPN Sst ablated animals showed increased time of center exploration in an open eld test (Supplementary Fig. 11l) without altering locomotor activity in this assay (Supplementary Fig. 11m).

## Discussion

Defensive behaviors are regulated by adaptive mechanisms contingent on the previous experience of an aversive outcome. Here we de ne early and late threat-evoked defensive responses that adjust to repeated VLS exposures when there is no evidence of real harm. We identify the GABAergic population in the IPN of the midbrain as a critical node orchestrating adaptive defensive strategies. Inhibitory projections from the IPN to the LDTg control the learning aspect of threat processing. In contrast, a subpopulation of IPN neurons expressing the neuropeptide Sst mediate generalized aspects of avoidance-related behaviors. The present findings have important implications for understanding the neurobiology of innate fear behaviors and how their expression is regulated in the absence of danger, a critical concept in many neuropsychiatric conditions.

The selection of ongoing defensive behaviors is constantly updated by recent experiences that contribute to perceptual and value-based decision-making and action selection (38, 39). While adaptations of defensive responses to visual looming stimuli have been reported in various animal species (38, 40–42), the behavioral signatures of threat adaptation and the circuit mechanisms underlying these behavioral changes remain largely unknown. We found that upon repeated VLS sessions, mice reduced immediate freezing, as well as late defensive behaviors including time spent inside the nest. Instead, animals switched behavioral strategies to engage in more foraging behavior. Importantly, changes in defensive strategies were detected over multiple-day sessions but not within a trial session, supporting the view that these may reflect learning and consolidation processes (38). Although most adaptive defensive behaviors to visual looming have been focused on escape responses (38, 40), understanding late emotional components of threat processing has received less attention. Our behavioral analysis revealed a dissociation between time spent inside the nest and escape responses over repeated VLS sessions and learning. Even though multiple defensive behaviors adjust with consecutive threat exposures that have no aversive outcome, they may each require differential circuitry to guide the behavioral output. Escape is a flexible behavior under cognitive control (39). Integrating variables of escape behavior together with the engagement of foraging strategies upon threat assessment -for instance when animals leave the nest to take new risks-, is necessary to understand how these are coordinated to drive adaptive behaviors. Given that the speed of habituation to VLS depends on the behavioral context (42) and it is stimulus-specific (38), future studies could help elucidate early and late defensive components of threat adaptation specific to the context or type of threatening stimuli.

The neural basis of threat detection and response to VLS are conserved across species (1, 7). Emerging evidence indicates that divergent circuits orchestrate escape and freezing responses to looming stimuli (2, 6, 43, 44). Yet, most of these studies have focused on a single looming session and thus, the neural circuitry of innate defensive adaptive learning to VLS remains unexplored. Here we provide first-time evidence that IPN GABAergic neurons are recruited by innate visual threats and show neuronal adaptation with defensive learning. These results support that the habenular axis is involved in anxiety (14–21) and fear-related behaviors (22–25), as well as familiarity signaling (30). Silencing overall IPN GABAergic activity during VLS presentations devalued the stress component of a VLS threat and reduced early freezing responses and late emotional behaviors such as time spent inside the nest. In contrast to the ventral tegmental area (VTA) GABAergic neurons (45), brief optogenetic stimulation of IPN GABAergic subpopulation was not sufficient to trigger early or late defensive behaviors, indicating specialized roles of VTA and IPN inhibitory networks.

Here, we report for the first time that activity dynamics of IPN GABAergic neurons inversely mirrored changes in speed. The IPN is part of the nucleus incertus network that controls locomotor speed, arousal, and hippocampal theta rhythms (46). Through this circuit connectivity, the IPN may contribute to the animals’ arousal and locomotion. Still, our findings showing reduced IPN GABAergic neuronal activity with nest entries -when animals lower speed-, suggest a role of the IPN beyond controlling locomotion to also processing affective behaviors like avoidance or approach. Freezing is cardinal in stress-coping processes as it corresponds to a state of hypervigilance that enables decision-making (2). Spending time inside the nest upon VLS could also represent a stress-coping mechanism that prepares for future foraging actions. Altogether, our results support the view that the IPN responds to aversive stimuli to regulate distinctive stress-related coping strategies (34).

IPN GABAergic neurons send strong inhibitory projections to the raphe and tegmentum (26, 27). Through the LDTg, the IPN controls nicotine aversion (29) or social novelty preference (28). We found IPN◊LDTg circuit engagement with aversive VLS and neuronal adaptation with multiple exposures. Optogenetic silencing the IPN◊LDTg circuit increased freezing and occluded late defensive adaptive learning specifically for time spent in shelter. Recent findings demonstrate LDTg GABAergic neurons inhibit VTA to promote unconditioned freezing responses (47). Although work from our group and others suggest IPN GABAergic neurons inhibit LDTg cholinergic neurons innervating the VTA (28, 29), we cannot exclude the possibility the IPN inhibits LDTg GABAergic function to regulate fear and freezing responses. Considering different LDTg interneurons oppositely regulate innate fear (35), understanding how the IPN controls LDTg function and network connectivity is necessary to elucidate the critical role of this circuit in innate defensive adaptive learning.

Among all IPN GABAergic cell types, we recently showed the neuronal population expressing the neuropeptide Sst is activated by acute stress to drive motivational behaviors (34). Sst provides retrograde inhibition of excitatory inputs from the mHb to the IPN (48). Our ber photometry data revealed engagement of IPN Sst neurons during threatening VLS events but also when animals entered the safety of a protective shelter. In the lateral habenula, heterogeneous neuronal clusters predict the selection of distinct threat-driven defensive behaviors (8). It could be possible that different IPN Sst populations exist to control various aspects of threat aversion vs. coping mechanisms. We show that genetic ablation of IPN Sst neurons reduced time inside the nest as well as general anxiety-like behaviors. Ablated animals did not recognize the nest as a safe, protective zone. Sst neurons can mediate anxiety by disinhibition of fear-related amygdaloid circuits (37). Elucidating the precise IPN Sst neuronal connectivity with previously established anxiety networks should be addressed in future investigations. Collectively, our findings implicate the IPN as a critical node of innate threat processing and adaptive defensive learning, suggesting dysregulation of the IPN may contribute to numerous psychiatric disorders.

## Data Availability

All data will be available upon request to the corresponding author.
